# Pregnancy Outcomes and Child Development Effects of SARS-CoV-2 Infection (PROUDEST Trial): Protocol for a Multicenter, Prospective Cohort Study

**DOI:** 10.2196/26477

**Published:** 2021-04-20

**Authors:** Geraldo Magela Fernandes, Felipe Motta, Lizandra Moura Paravidine Sasaki, Ângelo Pereira Da Silva, Andreza Monforte Miranda, Aleida Oliveira De Carvalho, Ana Paula Monteiro Gomides, Alexandre Anderson De Sousa Munhoz Soares, Agenor De Castro Moreira Dos Santos Jr, Caroline De Oliveira Alves, Ciro Martins Gomes, Clara Correia De Siracusa, David Alves De Araújo Jr, Dayde Lane Mendonça-Silva, José Alfredo Lacerda De Jesus, Karina Nascimento Costa, Maria Eduarda Canellas De Castro, Patricia Shu Kurizky, Paulo Sérgio França, Rosana Tristão, Yacara Ribeiro Pereira, Luiz Claudio Gonçalves De Castro, Alberto Moreno Zaconeta, Cleandro Pires De Albuquerque, Licia Maria Henrique Da Mota

**Affiliations:** 1 Faculty of Medicine University of Brasília Brasília - DF Brazil; 2 Department of Rheumatology University Hospital of Brasília Brasília Brazil; 3 State Health Department of the Federal District Brasília Brazil; 4 Faculty of Medicine University Center of Brasília Brasília Brazil; 5 Central Laboratory of Public Health of the Federal District Brasília Brazil

**Keywords:** SARS-CoV-2, COVID-19, pregnancy, neonate, children, outcome, development, prospective, cohort, women, fetus, baby, implication

## Abstract

**Background:**

A growing body of evidence suggests that SARS-COV-2 infection during pregnancy may affect maternal-fetal outcomes and possibly result in implications for the long-term development of SARS-CoV-2–exposed children.

**Objective:**

The PROUDEST (Pregnancy Outcomes and Child Development Effects of SARS-CoV-2 Infection Study) is a multicenter, prospective cohort study designed to elucidate the repercussions of COVID-19 for the global health of mothers and their children.

**Methods:**

The PROUDEST trial comprises 2 prospective, sequential substudies. The *PREGNANT* substudy will clinically assess the effects of SARS-CoV-2 infection on pregnancy, childbirth, and puerperium from a mechanistic standpoint to elucidate the pregnancy-related inflammatory and immunological phenomena underlying COVID-19. Pregnant women aged 18-40 years who have been exposed (proven with laboratory tests) to SARS-CoV-2 (group A; n=300) will be compared to control subjects with no laboratory evidence of in-pregnancy exposure to the virus (group B; n=300). Subjects exposed to other infections during pregnancy will be excluded. The *BORN* substudy is a long-term follow-up study that will assess the offspring of women who enrolled in the prior substudy. It will describe the effects of SARS-CoV-2 exposure during pregnancy on children’s growth, neurodevelopment, and metabolism from birth up to 5 years of age. It includes two comparison groups; group A (exposed; n=300) comprises children born from SARS-CoV-2–exposed pregnancies, and group B (controls; n=300) comprises children born from nonexposed mothers.

**Results:**

Recruitment began in July 2020, and as of January 2021, 260 pregnant women who were infected with SARS-CoV-2 during pregnancy and 160 newborns have been included in the study. Data analysis is scheduled to start after all data are collected.

**Conclusions:**

Upon completion of the study, we expect to have comprehensive data that will provide a better understanding of the effects of SARS-CoV-2 infection and related inflammatory and immunological processes on pregnancy, puerperium, and infancy. Our findings will inform clinical decisions regarding the care of SARS-CoV-2–exposed mothers and children and support the development of evidence-based public health policies.

**Trial Registration:**

Brazilian Register of Clinical Trials RBR65QXS2; https://ensaiosclinicos.gov.br/rg/RBR-65qxs2

**International Registered Report Identifier (IRRID):**

DERR1-10.2196/26477

## Introduction

### Background

The natural history of COVID-19, a disease caused by SARS-CoV-2, is still being written. Individuals infected with SARS-CoV-2 may present with a broad spectrum of clinical manifestations, from no symptoms to dramatically progressive disease symptoms that eventually result in death [1,2]. Some individuals develop intense inflammatory and procoagulant responses that can result in severe pulmonary damage, which is the main cause of COVID-19 morbidity and mortality.

The pathophysiological phenomena that take place in other human tissues that are potentially targeted by SARS-CoV-2 need further investigation. A central nervous system viral tropism has been postulated based on reports of neurological events such as stroke, acute hemorrhagic encephalopathy, seizures, and the loss of smell and taste [3,4].

To date, little is known about the effects of COVID-19 on women during pregnancy and puerperium [5,6] and its consequences for women’s offspring (from the neonatal period through the first years of life) [7-9]. Thus, there is still a lack of a robust evidence base for the proper management of these mothers and children.

Data from previous epidemics of viral-induced respiratory distress syndrome have shown that pregnant and puerperal women have a high risk of developing life-threatening clinical outcomes [10]. These generally worse outcomes have been attributed to physiological changes in the immune and cardiopulmonary systems that occur during pregnancy [11]. Examples of epidemics include the H1N1 influenza, SARS-CoV (severe acute respiratory syndrome coronavirus), and MERS-CoV (Middle East respiratory syndrome coronavirus) epidemics, during which pregnancy mortality rates reached 27% [10,12,13].

Despite the structural similarities between coronaviruses, the initial reports for pregnant women with COVID-19 showed lower rates of intensive care unit admission, orotracheal intubation, and death during the SARS-CoV-2 outbreak than those during the SARS-CoV and MERS-CoV outbreaks [9,13]. However, more recent papers have shown higher morbidity and mortality rates among pregnant women than those among nonpregnant women. Several aspects of embryo implantation [14], placental development [15], and delivery dynamics [16] seem to be impaired by the inflammatory response driven by immune cell subtypes at the maternal-fetal interface [17]. Such phenomena may precipitate preeclampsia, spontaneous abortion, intrauterine growth restriction, and premature birth [18-21].

Most of the available data on pregnant women exposed to SARS-CoV-2 were obtained during the second half of pregnancy. Thus, SARS-CoV-2 infection during all stages of pregnancy, including the early stages of gestation, has not been fully investigated. Nevertheless, as the disease spreads worldwide, more women are being exposed to the virus during early gestation and midgestation, and new data have been accruing [22].

Studies evaluating the vertical transmission of SARS-CoV-2 are still inconclusive [6,23-25]. Investigations of placentas from women infected with SARS-CoV-2 have suggested that there is a low likelihood of viral transplacental transmission. However, the potentially hazardous effects of inflammatory and prothrombotic environments on placental function and, consequently, fetal growth could not be ruled out [26-28].

To date, the few reports on postnatally infected neonates have shown that they exhibit either no symptoms or mild clinical forms of COVID-19 with favorable outcomes. However, the younger the infant is, the higher the risk of critical outcomes [22,29].

Maternal SARS-CoV-2 infection would potentially expose a fetus not only to direct viral effects but also to the placental inflammatory response and the maternal cytokine storm [5,7,22]. Such processes and their consequences have not been extensively studied. The understanding of these phenomena should contribute to the proper management of children born to mothers infected with SARS-CoV-2.

A case series on the clinical aspects of newborns of COVID-19–exposed mothers reported a low risk of adverse outcomes for late pregnancy exposure and stated that there is a paramount need for close follow-ups [30]. Other reports have presented data showing no adverse effects on neonates born to mothers who tested positive for COVID-19. Furthermore, Liu et al [31] described 19 completely asymptomatic neonates from Wuhan, China.

A few studies however have reported that SARS-CoV-2 test–negative neonates born to mothers who tested positive and developed critical illnesses might present ominous clinical profiles. This is suggestive of the potential impact of inflammatory processes on fetal physiology. Romagano et al [32] reported a prevalence rate of 6.9% for symptomatic pregnant women infected with SARS-CoV-2 among 1053 deliveries at a large hospital network in New Jersey, United States. They reported that 8 pregnant women were critically ill and 7 neonates tested negative (via reverse transcriptase–polymerase chain reaction [RT-PCR]); 1 neonate was not yet delivered at the time of testing. All neonates were preterm and appropriately sized for their gestational age except for one (small for their gestational age). They were all separated from their mothers after delivery, and all of them developed respiratory distress and required neonatal intensive care unit admission. Anemia and hyperbilirubinemia of prematurity, temperature instability, and feeding problems were reported in some of the neonates.

Several other studies have reported symptoms among SARS-CoV-2 test–negative neonates born to mothers with COVID-19, such as rashes [33], facial ulceration [33], the need for noninvasive oxygen support [33], transient lymphocytopenia [34], impaired liver function [34], disseminated intravascular coagulation, and even multiple organ failure leading to death [35]. There are many critical questions regarding the standards of care for SARS-CoV-2–exposed pregnant women and their offspring that have yet to be answered, and guidelines are still being developed around the world [8,36,37]. Therefore, the overall purpose of this study is to describe the effects of in-pregnancy SARS-CoV-2 infection and related inflammatory and immunological phenomena on the health of SARS-CoV-2–exposed women and their offspring.

### Objective

Our specific aims are (1) to study the effects of COVID-19 on maternal and obstetric morbidity and mortality, including those of indicators such as preeclampsia, abortion, fetal malformation, fetal growth, and premature birth; (2) to investigate the presence of SARS-CoV-2 and anti–SARS-CoV-2 antibodies in the cerebrospinal fluid (CSF) of women with symptomatic COVID-19 undergoing spinal anesthesia for a cesarean section; (3) to determine the serum proinflammatory and regulatory cytokine profiles of pregnant women with symptomatic COVID-19; (4) to determine the CSF proinflammatory and regulatory cytokine profiles of pregnant women with symptomatic COVID-19 undergoing spinal anesthesia for a cesarean section; (5) to study the histopathological markers of inflammatory and thrombotic phenomena in the placenta; (6) to study the correlations between the aforementioned serum and histologic biomarkers of COVID-19 and the outcomes of pregnancy, delivery, puerperium, and childbirth as well as the correlations between biomarkers and short- and long-term health outcomes during infancy; (7) to study the association between the use of maternal pharmacological therapy for treating COVID-19 and offsprings’ health outcomes; (8) to evaluate the effects of COVID-19 exposure during different stages of pregnancy on fetal, neonatal, and infantile morbidity and mortality; and (9) to evaluate the effects of in-pregnancy COVID-19 exposure on children’s somatic and neurological development and energy metabolism from birth up to 5 years of age.

## Methods

### Study Design

**Figure 1:**
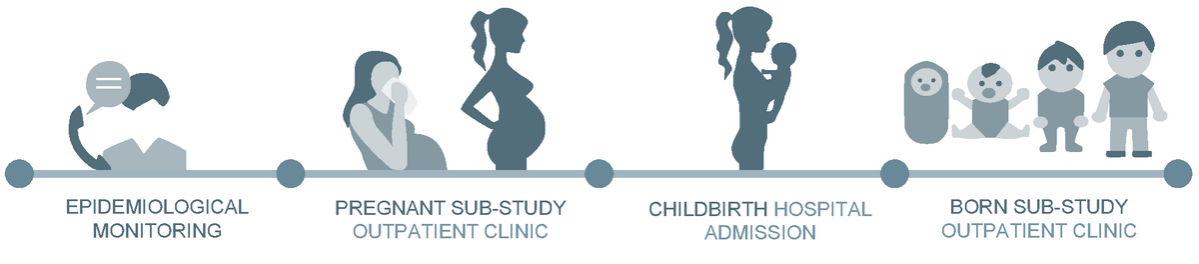
Pregnancy Outcomes and Child Development Effects of SARS-CoV-2 Infection Study follow-up flowchart.

The PROUDEST (Pregnancy Outcomes and Child Development Effects of SARS-CoV-2 Infection Study) is a multicenter, longitudinal, prospective observational study that will be conducted in two sequential stages—the *PREGNANT* and *BORN* branches (or substudies). Each stage will have two parallel groups (exposed and nonexposed) for comparisons. The PROUDEST is designed to address the multifaceted questions surrounding the impact of COVID-19 exposure during pregnancy on the global health of mother-child dyads (Figure 1).

The PREGNANT substudy will follow—until day 21 postpartum—pregnant women who are exposed to SARS-CoV-2 at any phase of gestation and compare them to a control group consisting of nonexposed pregnant women. The BORN substudy will follow the children of the women included in the preceding (PREGNANT) branch. These children will be allocated into two comparison groups (exposed and nonexposed) according to their mothers’ in-pregnancy exposure status and will be followed up by a multidisciplinary team of health professionals from birth up to the age of 5 years. This team will conduct regular consultations every month up until the children reach 6 months of age, every 3 months up until the children reach 2 years of age, and every 6 months up until the children reach 5 years of age. Mothers and children may attend nonscheduled visits, as needed, for clinical reasons as well as specific appointments for conducting the procedures and tests described in this protocol.

The PROUDEST will be conducted from July 2020 to December 2026 in Brasília, Brazil. The recruitment of pregnant and newborn dyads will be carried out by using data from the Epidemiological Surveillance Center of the Federal District. These mother-fetus dyads will be followed up to childbirth (and puerperium in the case of mothers) until December 2021, which is when the last included dyads are expected to undergo childbirth in two different hospitals—the University Hospital of Brasília and Asa Norte Regional Hospital (the reference public medical center for COVID-19 in the Federal District in Brazil). Both hospitals are located in central urban areas and are included in the Brazilian public health system (Sistema Único de Saúde), which primarily serves the low-income population. Thus, the results of the PREGNANT substudy will be published as soon as the analyses are completed. The children will be followed from childbirth up to December 2026, which is when the last admitted neonate will turn 5 years old. As the BORN substudy is lengthy, partial results may be disclosed during the course of the study, but the final data set will be made available in the second half of 2026.

Pregnant women included in the study must be aged >18 years. COVID-19 exposure will be defined as a first-time RT-PCR test, serology test, or rapid test that returns positive results during pregnancy and is confirmed by a second test. Nonexposure to COVID-19 will be defined as asymptomatic pregnant women with negative serology tests (immunoglobulin G [IgG] and immunoglobulin M [IgM] tests), which will be conducted at 14-21 days postpartum.

Pregnant women with preexisting chronic diseases (except diabetes and hypertension); those taking continuous medications; those who consume tobacco, alcohol, or other drugs; and those with other suspected or confirmed congenital infections will be excluded.

Neonates whose mothers qualified for inclusion in the PREGNANT substudy (had these women been screened) may also be admitted to the BORN substudy, even if their mothers did not participate in the preceding branch.

Children initially assigned to the control (nonexposed) group who later become infected with SARS-CoV-2 (as confirmed via laboratory tests) during follow-up will be excluded from all analyses (from the time of SARS-CoV-2 infection diagnosis onward). However, they will continue to receive assistance under the same standards until the end of the study.

### Sample Size Calculation

No precise data are available on the prevalence of SARS-CoV-2 infection among pregnant women in Brazil, but international reports have estimated that up to 15.3% of all pregnancies have been exposed to the virus [38]. Recent data have indicated a birth rate of 44,195 newborns per year in the Federal District [39]. Thus, after considering an “infinite” population (>20,000 pregnant women), assuming a 15% prevalence of SARS-CoV-2–exposed pregnancies, and accounting for a confidence level of 95% and a margin of error of 5%, the minimum sample size for a random sample of SARS-CoV-2–exposed women would be 195. This calculation expectedly yielded a similar number for a random sample of SARS-CoV-2–exposed children. If we set the expected dropout rate for the BORN substudy to 20%, the required number of SARS-CoV-2–exposed mothers (those giving birth to the BORN participants) would increase to 234.

Our sampling approach however is not truly random; it is based on convenience, as eligible subjects will present to the recruitment centers. The aforementioned calculations only serve as a reference for avoiding the overestimation of the inclusion of participants. Given the limited amount of available knowledge regarding the effects of SARS-CoV-2 infection on pregnancy and child development, which resulted in the eminently exploratory character of our study, we adopted an “as much as feasible, but no more than reasonable” approach for defining the sample size.

We set an a priori number of 300 SARS-CoV-2–exposed women for the PREGNANT phase. This will result in the inclusion of the expected 300 SARS-CoV-2–exposed children in the BORN phase. The subject allocation rate between the exposed and control groups was 1:1. This indicated the need for an additional 300 mothers and 300 children to constitute the nonexposed control groups. Hence, the overall sample size of the PROUDEST was set to 1200 participants (600 mother-child dyads consisting of 300 SARS-CoV-2–exposed mother-child dyads and 300 control dyads).

To promote participant retention and completed follow-ups at pregnancy and pediatric outpatient clinics, we will actively search for patients via phone and email.

### Procedures

**Figure 2:**
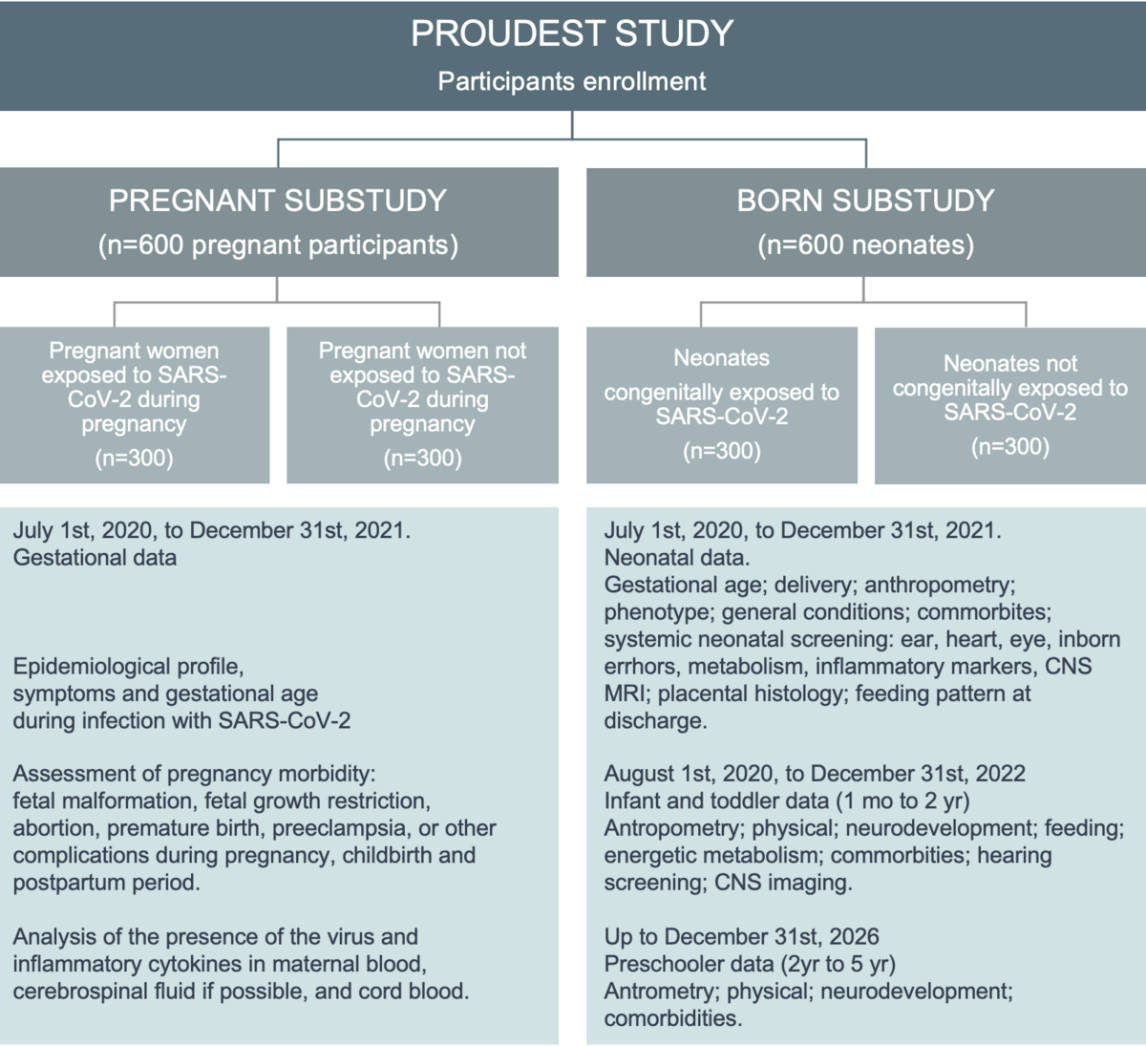
PROUDEST study design. CNS: central nervous system; MRI: magnetic resonance imaging; PROUDEST: Pregnancy Outcomes and Child Development Effects of SARS-CoV-2 Infection Study.

A host of clinical, psychological, neurodevelopmental, biochemical, histological, and imaging assessments will be conducted in accordance with the PROUDEST protocol (Figure 2).

Prenatal data from both the pregnant women and their fetuses will be gathered during the follow-up at the Pregnancy Outpatient Clinic of the University Hospital of Brasilia. These data will consist of the medical and sociodemographic data of the mothers; gestational age; symptoms, interventions, and outcomes related to COVID-19 (for SARS-CoV-2–exposed participants); congenital infection screening results; hypertensive disorders and other pregnancy-specific morbidities; general health assessments; general physical examinations; routine clinical biochemistry tests; and ultrasound scans. These scans will be performed between gestational weeks 11-13 and gestational day 6, from gestational week 22 to gestational week 24, and on a monthly basis in the third trimester of pregnancy to assess fetal growth and morphology, placental morphology, amniotic fluid volume, and dopplerfluxometry results. Maternal blood will be collected at the first prenatal consultation, regardless of gestational age. Antenatal consultations will occur monthly up to week 34, every 2 weeks between weeks 34 and 36, and then weekly up to delivery. During pregnancy, psychological risk assessments and mental health screens will be performed with the Beck Depression Inventory during the first prenatal consultation. [40]. Individual psychological care will be provided to pregnant women who have a Beck Depression Inventory score of >12. Mothers will also be physically and psychologically evaluated between days 7 and 21 postpartum during the PREGNANT substudy and after the BORN substudy.

At childbirth, assessments will be conducted to identify the occurrence of dysfunctional labor and the premature rupture of membranes, the type of birth, and delivery outcomes. We will also conduct physical examinations and classifications of the newborns and anthropometry. The early initiation of breastfeeding, the need for neonatal intensive care, and the type of interventions will also be identified. Maternal blood, CSF from women undergoing spinal anesthesia for a cesarean section, and umbilical cord blood samples will be collected.

CSF will be collected immediately before the infusion of the medicine for spinal anesthesia, which will be injected via a sterile syringe at an average dosage of 0.5 ml. CSF will be collected in a 4-mL cryotube. Blood samples from mothers and the umbilical cord will be collected in a heparinized tube and centrifuged immediately, and the plasma will be stored in 4-mL sealed cryotubes. CSF and blood samples will be subsequently stored at −80 °C for later analysis. The assessment of blood cell counts, inflammation markers (C-reactive protein and procalcitonin), biochemistry (alanine aminotransferase, aspartate aminotransferase, ferritin, alkaline phosphatase, and lactic dehydrogenase) and SARS-CoV-2 tests (RT-PCR and IgM and IgG antibody tests) will be carried out.

Circulating cytokine levels will be evaluated with the Luminex Bio-Plex Pro Human Cytokine 27 platform (Bio-Rad Laboratories). The cytokine profile assessment will analyze chemokines (CXC motif chemokine ligand [CXCL] 8, CC motif chemokine ligand [CCL] 11, CCL3, CCL4, CCL2, CCL5, and CXCL10), proinflammatory cytokines (interleukin [IL]-1β, IL-6, tumor necrosis factor, IL-12p70, interferon γ, IL-17A, and IL-15), regulatory cytokines (IL-1Ra, IL-4, IL-5, IL-9, IL-10, and IL-13), and cell growth factors (IL-2, IL-7, basic fibroblast growth factor, platelet-derived growth factor, vascular endothelial growth factor, granulocyte colony-stimulating factor, and granulocyte-macrophage colony-stimulating factor). All procedures will be performed according to the manufacturer's recommendations.

Analyses will be performed at the clinical biochemistry laboratories of the hospitals where delivery occurred (blood cell count, C-reactive protein, procalcitonin, and routine biochemistry assessments), the Central Laboratory of the Federal District Department of Health (Laboratório Central de Saúde Pública; SARS-CoV-2 tests), and the University of Brasilia laboratory (cytokine profile assessments).

The placenta will be subjected to fresh histopathological analyses for assessing possible morphological and histological changes that may be associated with SARS-CoV2 infection. Histopathological analysis will be conducted according to the Amsterdam protocol [41].

Peripheral blood and CSF samples from the newborns will only be collected if there is clinical need; this will not be done per the routine research protocol. If such specimens are made available, they will also be subjected to the aforementioned analyses.

All newborns will undergo neonatal screening tests in accordance with the recommendations of the Ministry of Health of Brazil. Five drops of blood will be collected on filter paper for the national neonatal screening program. This blood sample will be collected after 48 hours of life and will be used to screen for the following diseases: phenylketonuria, congenital hypothyroidism, biotinidase deficiency, cystic fibrosis, and congenital adrenal hyperplasia. After 24 hours of life and before discharge, a pulse oximetry test will be performed. Oxygen saturation in the right upper limb and one of the lower limbs will be measured. The pulse oximetry test will be considered normal if oxygen saturation is ≥95% and the difference between the limbs is not ≥3%. Hearing screening will be performed between 36 and 48 hours of life by analyzing otoacoustic emissions.

After hospital discharge, all neonates will be followed up at the Pediatric Outpatient Clinic of the University Hospital of Brasilia. Child growth and neurodevelopment will be assessed at all visits. The first visit will be scheduled to occur on day 15 postpartum. Afterward, visits will be conducted monthly during the first 6 months of life. Thereafter, visits will be scheduled every 3 months until the children reach 12 months of age and every 6 months until the children reach 5 years of age. Nonscheduled visits may occur due to urgent clinical needs. The outpatient clinic staff (a multidisciplinary team) will be composed of pediatricians, psychologists, occupational therapists, speech therapists, physiotherapists, and nurses. The psychological effects of SARS-CoV-2 infection on mothers will be assessed with the Edinburgh Postnatal Depression Scale [42]. This assessment will occur more than once until their children reach 6 months of age. Breastfeeding and weaning patterns, dietary habits, nutritional status, and vaccinal status will be assessed throughout the study.

The assessment of children’s neurodevelopment will be carried out until they reach the 60th month of life. This assessment will analyze cognitive, motor, socioemotional, and language-related aspects and adaptive behavior. The evaluation will be conducted by using the Bayley III Child Development Scale (ie, the version validated for Brazilian infants) [43]. From the age of 2.5 years onward, aspects related to intellectual performance will also be assessed by using the Wechsler Preschool and Primary Intelligence Scale, third edition at 6-month intervals [44].

Central nervous system imaging assessments will be carried out via transcranial ultrasound doppler scans, which will be performed between the 15th and 90th day of the child's life. A brain magnetic resonance imaging scan will be performed if altered cephalic perimeter measures, neurological development delays, or abnormal ultrasound doppler scan findings are identified.

Blood will be collected from SARS-CoV-2–exposed children aged 12 and 24 months to assess their metabolic profiles, which will be used to identify the long-term effects of SARS-CoV-2 infection on systemic metabolism that are potentially driven by past viral exposure and associated inflammatory responses. The examination will consist of assessments for energy metabolism markers (serum lipids, glucose, and insulin), thyroid function markers (thyroid-stimulating hormone and free thyroxine), bone metabolism markers (parathyroid hormone, calcium, phosphate, alkaline phosphatase, and 25-hydroxyvitamin D), adrenal tonus indicators (adrenocorticotropic hormone and basal cortisol), and renal function markers (blood urea nitrogen, creatinine, and urine analysis results).

All newborns will undergo extended hearing screening. Evoked otoacoustic emission and brainstem auditory-evoked potential tests will be performed during the child’s first year of life.

### Statistical Analysis

All data will be stored in REDCap (Research Electronic Data Capture; Vanderbilt University), which is a tool for building and managing web-based surveys and databases. All variables will be summarized via standard descriptive techniques according to their type and distribution. For the bivariate analysis, differences in categorical variables between the exposed and unexposed groups will be verified with the Chi-square test or Fisher exact test, whereas differences in continuous variables between the groups will be assessed with the Student *t* test or the Mann-Whitney U test.

For dichotomous outcomes, binomial regression models, which will be adjusted based on the unbalanced and relevant background features of the comparison groups, will be used to estimate the relative risks between the exposed and nonexposed groups. Partial correlation and general linear models will be used to assess the associations between continuous outcome variables and covariates; adjustments for imbalances will be made as appropriate. A *P* value of <.05 will be considered significant. The control group will be composed of nonexposed mother-child dyads that meet the inclusion criteria. However, those with positive serology tests (IgG and IgM tests conducted at 14-21 days postpartum) will not be included in the control group. The groups will not be matched or paired based on age or other variables. However, any differences between the groups will be adjusted later via statistical means.

### Ethics Approval and Consent to Participate

The PROUDEST was approved by the Research Ethics Committee of the University of Brasilia School of Medicine (Certificado de Apresentação de Apreciação Ética 32359620.0.0000.5558) [45]. It was also registered in the Brazilian Register of Clinical Trials [46]. All pregnant women participating in the PROUDEST are required to sign an informed consent form to join the PREGNANT branch. Likewise, the participation of the children in the BORN branch will require signed, informed consent from their mothers. The 6-month reports on the study’s status and its partial results will be made available to the institutional Research Ethics Committee and may be publicly consulted upon request.

### Availability of Data and Materials

At the time of the publication of this protocol, study enrollment and data collection have already started, but we have not completed the participant recruitment and data analysis phases. Therefore, data sharing is not yet feasible, as no data sets have been generated or analyzed at this stage of the study. As partial data become available, they will be displayed in the Brazilian Register of Clinical Trials [46].

## Results

The PROUDEST is in the data collection phase. Study recruitment started in July 2020. As of January 2021, a total of 260 pregnant women who were infected with SARS-CoV-2 during pregnancy and 180 newborns from hospitals in the Federal District in Brazil have been included in the study. Data analysis is scheduled to start after all data are collected.

## Discussion

### Study Implications

The PROUDEST offers comprehensive insight (from both the obstetric and pediatric perspectives) into the effects of SARS-CoV-2 infection on the global health of pregnant women and their offspring. Specifically, the study will fill the deep gap in knowledge about the consequences of SARS-CoV-2 infection during early gestation (ie, the period when the critical stages of embryogenesis take place), as women in all stages of pregnancy will be followed. The virus-induced inflammatory and immunological phenomena that occur in SARS-CoV-2–exposed mothers during this early period of life may have a particular impact on placental and fetal physiology or may even be associated with epigenetic signals. Therefore, these phenomena could conceivably affect the long-term outcomes of a child’s growth, development, and metabolism.

A better understanding of these potential long-term consequences requires lengthy, prospective observational studies, such as the PROUDEST. This study will not only address the clinical outcomes associated with in-pregnancy exposure to COVID-19 but also evaluate a host of soluble tissue biomarkers (as described in this protocol) with the aim of comprehensively understanding the mechanisms underlying related clinical phenomena.

Our data will add to the overall clinical and basic knowledge base for COVID-19, and our ultimate goal is to provide grounds for better managing SARS-CoV-2–exposed pregnant women and their children through direct means or by setting the stage for additional studies. In fact, the PROUDEST opens up a broad spectrum of possibilities for further, multidisciplinary research on the effects of SARS-CoV-2 infection on maternal, fetal, and pediatric health. Furthermore, our results might prove to be relevant from a social perspective, as they may provide data that support the tailored development and implementation of health policies that are specifically oriented to this particular demographic group.

The study does have several limitations. Its observational nature limits inferences for causal associations to some extent. However, the cohort study design is the closest observational equivalent to a clinical trial in terms of analytical power, and the objective of the PROUDEST does not ethically allow for interventional experiments because such experiments would imply that pregnant woman will be randomized based on SARS-CoV-2 infection. Moreover, the purpose of the study is to characterize the clinical and pathophysiological phenomena associated with SARS-CoV-2 infection during pregnancy and infancy, not to test the efficacy of any intervention. Thus, we believe that a cohort study is the best possible study design for addressing our objectives. The lack of random allocation for comparison groups will be partially compensated by statistically adjusting for the observed imbalances. Vaccination for SARS-CoV-2 will not be an exclusion criterion because due to the vaccine’s limited accessibility and availability, there are no feasible methods for estimating the proportion of pregnant women that will receive the vaccine. However, the effects of SARS-CoV-2 vaccination can be adjusted and analyzed in small control groups that have either received or not received the vaccine.

The protracted follow-up in the BORN substudy is expected to result in the dropout of several participants. We set a sample size for the study after taking into consideration a 20% loss to follow-up rate. This might ensure that a sizable number of children are available for the final assessment when they reach age of 5 years. However, we cannot avoid survival bias in the long-term data. Nevertheless, given that the continuity of multidisciplinary assistance will be guaranteed for all children in the BORN branch throughout the study period regardless of their withdrawal from the analysis or (temporary) losses to follow-up, we expect that children experiencing health problems associated with in-pregnancy COVID-19 exposure will be less likely to drop out than those who are in perfect health. Therefore, we do expect to have a sufficient number of children in the long run for identifying developmental abnormalities (should they exist), even after some amount of dropout.

Several routine pediatric consultations will be emphasized in the study, such as checking the kind of alimentation that a child is receiving (human milk or formula milk) and promoting the practice of breastfeeding.

### Conclusions

The PROUDEST is a long-term, prospective cohort study designed to provide a comprehensive analysis of the effects of in-pregnancy exposure to COVID-19 on women and their offspring from a clinical and pathophysiological standpoint. Our results might contribute to the improvement of the management of SARS-CoV-2–exposed mother-child dyads—through direct means or by setting the stage for future related studies—by providing knowledge on the clinical-pathophysiological phenomena associated with COVID-19 exposure among this particular population.

## Abbreviations

CCL: CC motif chemokine ligand
CNS: central nervous system
CSF: cerebrospinal fluid
CXCL: CXC motif chemokine ligand
IgG: immunoglobulin G
IgM: immunoglobulin M
IL: interleukin
MERS-CoV: Middle East respiratory syndrome coronavirus
PROUDEST: Pregnancy Outcomes and Child Development Effects of SARS-CoV-2 Infection Study
REDCap: Research Electronic Data Capture
RT-PCR: reverse transcriptase–polymerase chain reaction
SARS-CoV: severe acute respiratory syndrome coronavirus
